# Inactivation of *Lsd1* triggers senescence in trophoblast stem cells by induction of *Sirt4*

**DOI:** 10.1038/cddis.2017.48

**Published:** 2017-02-23

**Authors:** Josefina Castex, Dominica Willmann, Toufike Kanouni, Laura Arrigoni, Yan Li, Marcel Friedrich, Michael Schleicher, Simon Wöhrle, Mark Pearson, Norbert Kraut, Michaël Méret, Thomas Manke, Eric Metzger, Roland Schüle, Thomas Günther

**Affiliations:** 1 Urologische Klinik und Zentrale Klinische Forschung, Universitätsklinikum Freiburg, Medizinische Fakultät, Albert-Ludwigs-Universität Freiburg, Freiburg, Germany; 2 Celgene Quanticel Research, San Diego, CA, USA; 3 Max Planck Institute of Immunobiology and Epigenetics, Freiburg, Germany; 4 metaSysX GmbH, Potsdam-Golm, Germany; 5 Boehringer Ingelheim, Vienna, Austria; 6 Deutsches Konsortium für Translationale Krebsforschung, Standort Freiburg, Freiburg, Germany; 7 BIOSS Centre of Biological Signalling Studies, Albert-Ludwigs-University Freiburg, Freiburg, Germany

## Abstract

Coordination of energy metabolism is essential for homeostasis of stem cells, whereas an imbalance in energy homeostasis causes disease and accelerated aging. Here we show that deletion or enzymatic inactivation of lysine-specific demethylase 1 (Lsd1) triggers senescence in trophoblast stem cells (TSCs). Genome-wide transcriptional profiling of TSCs following Lsd1 inhibition shows gene set enrichment of aging and metabolic pathways. Consistently, global metabolomic and phenotypic analyses disclose an unbalanced redox status, decreased glutamine anaplerosis and mitochondrial function. Loss of homeostasis is caused by increased expression of *sirtuin 4* (*Sirt4),* a Lsd1-repressed direct target gene. Accordingly, Sirt4 overexpression in wild-type TSCs recapitulates the senescence phenotype initiated by Lsd1 deletion or inhibition. Inversely, absence of Lsd1 enzymatic activity concomitant with knockdown of *Sirt4* reestablishes normal glutamine anaplerosis, redox balance and mitochondrial function. In conclusion, by repression of Sirt4, Lsd1 directs the epigenetic control of TSC immortality via maintenance of metabolic flexibility.

Age-associated pathophysiology is caused by a progressive decline in regenerative capacities, which is unequivocally linked to a decreased potential of stem cells. The capability of stem cells to differentiate into specialized cell types allows for the replacement of damaged or deteriorated tissues. In addition, their capacity to proliferate is essential for self-preservation. Thus, understanding the molecular mechanisms for self-maintenance of stem cells is an important issue in the prevention of age-related diseases.^1^ Cellular senescence is a stress response that accompanies stable exit from the cell cycle without affecting cellular viability. The stress can be diverse such as telomere shortening, oncogene activation, reactive oxygen species (ROS), mitochondrial dysfunction or aberrant epigenetic modification.^2, 3, 4, 5, 6, 7^ The irreversible cease of proliferation in senescent cells is caused by increased expression of cell cycle inhibitors such as cyclin-dependent kinase inhibitor 2a (p16) and 2b (p15) and hypophosphorylation of retinoblastoma 1 (Rb) for the implementation of senescence.^6^ Finally, senescent cells are hallmarked by aberrant lysosomal *β*-galactosidase activity^8^ and can be identified by Rb-mediated chromatin-dense foci.^9^

Oxidative stress is mostly generated in mitochondria.^10^ Stem cells are particular sensitive to increased oxidants ^11^ suggesting a link between high ROS levels and a decline in stem cell number during aging.^12, 13^ The ability of cells to antagonize oxidative damage is determined by a controlled balance between the emergence of ROS and the capacity to generate antioxidants. The tripeptide glutathione (GSH) is the most abundant reducing agent within a cell, which can be oxidized to glutathione disulphide (GSSG) and regenerated to GSH at the expense of NADPH.^10^ The level of GSH declines during aging and disease resulting in elevated oxidative stress, but the underlying molecular mechanism remains elusive.^14, 15, 16^ GSH is synthesized from glutamate, cysteine and glycine, in which the conjunction of glutamate with cysteine delineates the rate-limiting step.^10, 17^ Glutamate is derived from glutamine, the most abundant free amino acid and the main nitrogen donor for synthesis of nucleotides and amino acids. Many cell types including cancer cells rely on glutaminolysis for maintenance of redox balance by generation of GSH and NADH and by replenishing tricarboxylic acid (TCA) cycle (glutamine anaplerosis) for biosynthesis and as energy source.^10^ A prominent antagonist of glutamine anaplerosis is sirtuin 4 (Sirt4), a member of the conserved NAD^+^-dependent protein deacetylase/ADP-ribosyltransferase family implicated in diverse biological processes.^18, 19^ Sirt4 is localized in the mitochondrial matrix^20^ and has been shown to restrain proton leakage, consequently increasing the membrane potential and reducing mitochondrial oxygen consumption.^21^ Independently, Sirt4 also controls glutamine anaplerosis by repression of glutamate dehydrogenase and glutamine uptake.^22, 23, 24^

Disclosure of the crosstalk between metabolic signals and a complex network of genetic and epigenetic pathways including histone methylation that direct self-renewal, potency, and differentiation of stem cells has just begun.^25, 26^ The first identified histone demethylase lysine-specific demethylase 1 (Lsd1; also called Kdm1a) can remove di- and monomethyl groups from histone H3 at lysine 4 (H3K4)^27, 28^ and lysine 9 (H3K9).^29, 30^ Ubiquitous deletion,^31, 32, 33, 34, 35, 36^ as well as deletion of *Lsd1* in trophectoderm or epiblast leads to early embryonic lethality.^36^ Lsd1 has been associated with repression of stem cell genes at the onset of mouse embryonic stem cell (ESC) differentiation, whereas it is dispensable for ESC maintenance.^37^ In contrast, in human ESCs LSD1 balances self-renewal and differentiation.^31^ In trophoblast stem cells (TSCs), giving rise to the embryonic part of the placenta, overexpressed Lsd1 has been reported to interact with oestrogen-related receptor *β*,^38^ a nuclear hormone receptor essential for placental formation.^39^ Deletion of *Lsd1* in TSCs skews cell fate and, despite the preservation of stemness marker gene expression in undifferentiated TSCs, leads to increased cell size^36^ indicating the requirement for Lsd1 to sustain proper stem cell characteristics. Furthermore, analyses of adipocytes implicate Lsd1 in the regulation of mitochondrial homeostasis.^40, 41, 42^ To date, little is known about the molecular mechanisms linking chromatin modification, metabolism and stem cell maintenance.

Here we show that loss, genetic or chemical inhibition of the histone demethylase Lsd1 triggers senescence in TSCs. Decrease of Lsd1 activity induces deregulated anaplerotic metabolism and loss of redox balance by elevated expression of the direct target gene *Sirt4*. Consequently, increased Sirt4 levels are sufficient to repress glutamine anaplerosis, disrupt redox homeostasis and induce senescence. *Vice versa*, reduction of Sirt4 expression in the absence of Lsd1 activity prevents premature senescence in TSCs. In summary, our data establish Lsd1-directed metabolism as a vital hub for the control of senescence in TSCs.

## Results

### Lsd1 deficiency boosts senescence in TSCs

**Figure 1:**
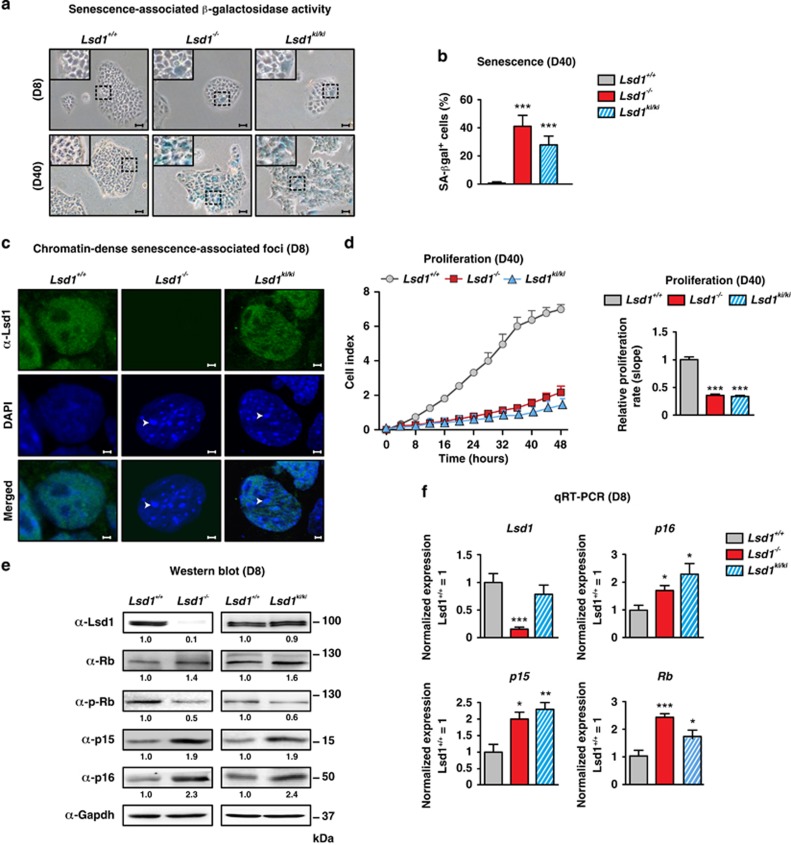
Lsd1 deficiency boosts senescence in TSCs. (**a**–**f**) Comparison of wild-type (*Lsd1*^+/+^), *Lsd1*-deficient (*Lsd1*^−/−^) and *Lsd1* enzymatic inactive mutant (*Lsd1*^*ki/ki*^) TSCs. (**a** and **b**) Representative images of senescent-associated *β*-galactosidase activity (blue) in TSCs (**a**) and quantification of senescent cells (**b**). (**c**) DNA stain by DAPI depicts chromatin-dense senescent-associated foci (arrows). TSCs were co-stained with an Lsd1 antibody. (**d**) Proliferation of TSCs was determined in real time. (**e**) Western blot decorated with the indicated antibodies including phosphorylated Rb (p-Rb). Band intensity was normalized to Gapdh as loading control relative to *Lsd1*^+/+^. (**f**) qRT-PCR of *Lsd1* and indicated cell cycle regulators normalized to three housekeeping genes relative to transcription in wild-type TSCs. Scale bars: 10 *μ*m (**a**) and 2 *μ*m (**c**). Data were analyzed from at least three biological samples and are represented as mean+S.E.M. *P<0.05; **P<0.01; ***P<0.001 (unpaired, two-tailed Student's *t*-test, (**b**, **d** and **f**))

We previously showed that deletion of *Lsd1* (*Lsd1*^−/−^) in TSCs^36^ leads to altered morphology reminiscent of senescent cells,^6^ which prompted us to inspect senescence-associated lysosomal hydrolase *β*-galactosidase activity. We observed a marked increase of *β*-galactosidase-positive stained cells within 40 days in *Lsd1*-deleted compared with wild-type (*Lsd1*^+/+^) cultures (Figures 1a and b). To examine any requirement for the enzymatic activity of Lsd1, we analyzed the impact of two different Lsd1-specific, nanomolar affinity inhibitors (Lsd1^i-1^; ORY-1001 (ref. 43); Lsd1^i-2^; QC6688) on senescence in wild-type TSCs. We observed a marked raise in the number of senescence-associated *β*-galactosidase-positive cells after 8 days of Lsd1 inhibition (Supplementary Figure S1a). In agreement, proliferation of TSCs incubated with Lsd1 inhibitors decreased in comparison with the control (Supplementary Figure S1b). To verify the impact of the demethylase activity of Lsd1 in controlling senescence, we generated TSCs homozygous for a conditional, enzymatically inactive mutant knockin allele. The endogenous Lsd1 gene is replaced with the mutant (*Lsd1*^ki/ki^) after induction of CreERT2-mediated recombination. Similar to *Lsd1*-deficient stem cells, the percentage of cells stained for senescence-associated *β*-galactosidase activity was robustly increased compared with wild-type TSCs within 40 days (Figures 1a and b). To corroborate premature senescence in TSCs devoid of Lsd1 activity, we stained cells with DAPI. In contrast to wild-type cells, senescence-associated nuclear foci were clearly visible in *Lsd1*^−/−^ and *Lsd1*^ki/ki^ TSCs (Figure 1c). Consistently, proliferation was severely impaired in *Lsd1*-deficient and *Lsd1* enzymatic inactive mutant TSCs in comparison with wild-type cells after 40 days (Figure 1d).

At the molecular level, comparison of control to *Lsd1*^−/−^ and *Lsd1*^ki/ki^ cells disclosed increased protein levels of Rb, p15 and p16, while phosphorylated Rb was reduced 8 days after addition of tamoxifen as indicated in Figure 1e. Quantification of p16 and phosphorylated Rb for individual cells by FACS in wild-type and *Lsd1*-deficient cells verified the altered protein levels as indicated by Supplementary Figure S1c. TSCs cultured in the presence of Lsd1 inhibitors for 24 h already displayed upregulation of cell cycle inhibitors (Supplementary Figure S1d), which was corroborated by quantitative reverse transcription PCR (qRT-PCR; Figure 1f and Supplementary Figure S1e). Furthermore, we depicted the dynamic of the phenotype by quantification of p16 and phosphorylated Rb at the single-cell level. TSCs cultured in the presence of Lsd1 inhibitor for 24 h, 3 and 8 days showed a progressive increase of p16 and a decrease of phosphorylated Rb immunoreactivity in comparison with control cells (Supplementary Figure S1f). Moreover, we also analyzed DNA damage by quantification of phospho γH2ax. The level was increased 8 days after *Lsd1* deletion and in TSCs inhibited for 24 h (Supplementary Figures S1g and h). As Lsd1 has been associated with DNA damage response,^44, 45, 46^ we also quantified p53, phosphorylated p53 and p21. Although we did not detect a direct impact of Lsd1 on transcription, we observed increased protein levels for p53, phospho 53 and p21 in accordance with elevated phospho γH2ax levels as indicated in Supplementary Figures S1g and h.

Taken together, our data demonstrate that the loss of Lsd1 demethylase activity, either by genetic deletion, chemical inhibition or replacement of enzymatic-active by enzymatic inactive Lsd1 inevitably provokes senescence in TSCs.

### Lsd1-controlled transcription regulates metabolic pathways and aging

**Figure 2:**
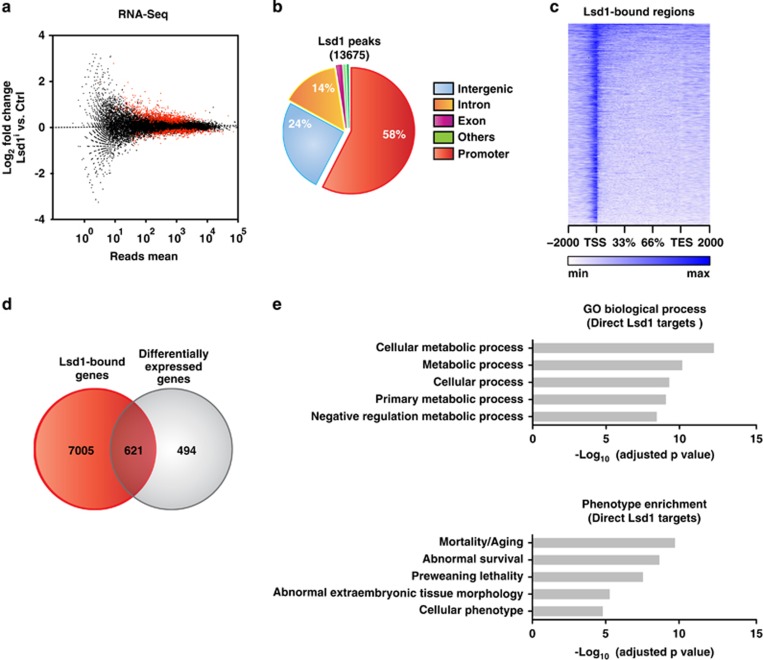
Lsd1-controlled transcription regulates metabolic pathways and aging. (**a**, **d** and **e**) Comparison of TSCs cultured for 24 h in the presence of solvent (Ctrl) or Lsd1 inhibitor 1 (Lsd1^i-1^) by RNA-sequencing (RNA-seq). (**a**) Significantly differentially transcribed genes with more than 50 reads are depicted as red dots. RNA-seq was done with two samples. (**b**) Pie chart displaying the genomic distribution of Lsd1 peaks in TSCs determined by ChIP-seq. ChIP-seq was performed with a single sample. (**c**) Read coverage for genomic Lsd1-binding sites in TSCs between transcription start (TSS) and transcription end site (TES) including 2 kb at the 5' and 3' site. (**d**) Direct Lsd1 targets were determined by intersection of Lsd1 promoter-occupied and differentially expressed genes. (**e**) Enrichment analyses for promoter-occupied and differentially expressed Lsd1 targets according to *P*-value. Statistical analysis was performed using MACS (ChIP-seq) and edgeR (RNA-seq)

To gain first insight into the molecular underpinnings of Lsd1-controlled senescence, we performed transcriptome analysis by RNA-sequencing (RNA-seq). We cultured wild-type TSCs in the presence or absence of a Lsd1-specific inhibitor for 24 h to unravel immediate early differentially expressed genes before senescence-associated *β*-galactosidase activity is increased (Supplementary Figure S1a). We obtained 1115 differentially expressed genes (≥1.4-fold expression change, ≥50 reads and *P*≤10^−5^) with on average 1.9-fold change in transcription (Figure 2a). To identify direct target genes of Lsd1, we also determined genome-wide Lsd1 chromatin occupancy in wild-type TSCs by chromatin immunoprecipitation using an Lsd1 antibody followed by massive parallel sequencing (ChIP-seq). The antibody has previously been validated and specifically recognizes Lsd1.^40^ In total, we identified 13 675 high confidence Lsd1 peaks with a false discovery rate (FDR) <0.1%. (Figure 2b). Lsd1-binding sites within the promoter were centred around the transcription start site (Figure 2c). Intersection of the Lsd1 promoter occupancy and transcriptome data sets revealed 621 differentially expressed direct target genes of Lsd1 in TSCs of which 59% were upregulated (Figure 2d; Supplementary Table S1). Enrichment analyses depicted metabolic processes and mortality/aging as the top-ranking categories for differentially expressed direct Lsd1 target genes (Figure 2e).

### Lsd1 regulates anaplerosis and maintains redox balance

**Figure 3:**
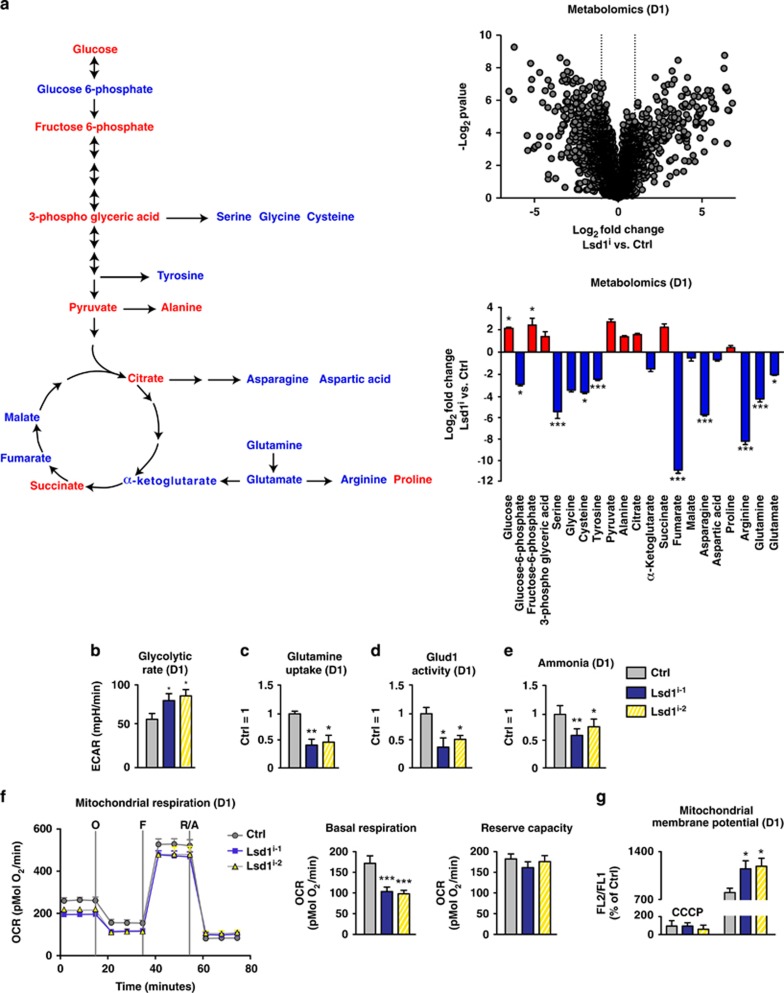

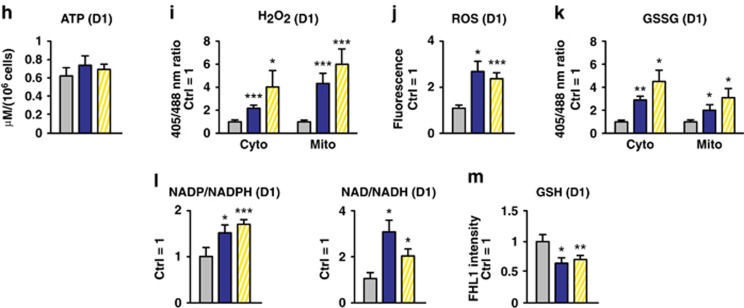
Lsd1 regulates anaplerosis and maintains redox balance. (**a**–**m**) Comparison of TSCs cultured for 24 h in the presence of solvent (Ctrl), Lsd1 inhibitor 1 (Lsd1^i-1^) (**a**–**m**), and Lsd1 inhibitor 2 (Lsd1^i-2^) (**b**–**m**). (**a**) Metabolomics profile depicted by log_2_ fold change *versus* −log_10_ *P*-value. Schema and bar graph depicting increased (red) and decreased (blue) metabolites of glycolysis, TCA cycle and glutamine anaplerosis. (**b**–**e**) Quantification of glycolysis rate by extracellular acidification rate (ECAR) (**b**), relative glutamine uptake (**c**), Glud1 activity (**d**) and ammonia levels (**e**). (**f**) Mitochondrial respiration was determined by a time course of OCR. Complex V was blocked by oligomycin (O), uncoupling was induced by FCCP (F) and electron transport system was disabled by addition of rotenone (R) and antimycin A (A). Basal respiration is the difference of the OCR in the absence of inhibitors and after addition of rotenone and antimycin A. The reserve capacity is derived from the subtraction of the OCR after addition of rotenone and antimycin A from oligomycin-treated TSCs. (**g**) Relative mitochondrial membrane potential determined by FL2/FL1 ratio. The background level was assigned by depolarization with CCCP. (**h**–**m**) Measurement of ATP concentration (**h**), relative H_2_O_2_ concentration determined with cytoplasmic and mitochondrial ratiometric reporters (**i**), relative quantification of ROS using fluorescent dye (**j**), relative concentration of oxidized glutathione (GSSG) determined by cytoplasmic and mitochondrial ratiometric reporters (**k**), relative ratio of oxidized to reduced NADP and NAD (**l**) and relative glutathione levels (**m**). Data were analyzed from at least three biological samples and are represented as mean+S.E.M. *P<0.05, **P<0.01, ***P<0.001 (unpaired, two-tailed Student's *t*-test)

To gain further insight into the dynamics of Lsd1-regulated metabolism, we quantified annotated metabolites of wild-type TSCs at two different time points by mass spectrometry (LC-MS/MS). We cultured TSCs in the presence or absence of Lsd1 inhibitor for 24 h and with or without tamoxifen for 8 days (Figure 3a and Supplementary Figure S2a). For metabolites of glycolysis, we observed an increase in glucose, fructose 6-phosphate and pyruvate after 24 h of inhibition and 8 days after Lsd1 deletion. Indeed, glycolysis measured by extracellular acidification rate (ECAR) was increased upon inhibition of Lsd1 for 24 h (Figure 3b) and was even further enhanced in *Lsd1*-deficient TSCs after 8 days (Supplementary Figure S2b). In contrast, glutamine anaplerosis was impaired reflected by a decrease in glutamine, glutamate and *α*-ketoglutarate levels (Figure 3a and Supplementary Figure S2a). Quantification of glutamine uptake by TSCs and glutamate dehydrogenase (Glud1) activity corroborated reduced glutamine anaplerosis (Figures 3c and d). As ammonia is mostly derived from glutamine anaplerosis,^47^ we also measured NH_4_^+^ concentration. The level of ammonia in TSCs was clearly decreased upon inhibition of Lsd1 (Figure 3e), which may explain why the levels of many amino acids were reduced (Figure 3a).

As the flux into the TCA cycle is directly linked to oxidative phosphorylation, we determined mitochondrial function in the presence or absence of Lsd1 activity after 24 h by quantification of the normalized oxygen consumption rate (OCR). We quantified the basal respiration by subtraction of the non-mitochondrial OCR after disabling the electron transport system by addition of rotenone (R) and antimycin A (A). The basal respiration was decreased in TSCs in the presence of Lsd1 inhibitor (Figure 3f). The reserve capacity depicts the capability of cells to respond to increased energy demand or stress. It is determined by subtracting the basal OCR from the maximal respiration after uncoupling by FCCP (F). The reserve capacity was not affected by Lsd1 inhibition (Figure 3f). Finally, we determined the mitochondrial membrane potential by JC-1 assay. Inhibition of Lsd1 for 24 h induced an increase in the membrane potential in comparison with the control (Figure 3g). Our data show that oxygen consumption was still coupled to emergence of ATP after Lsd1 inhibition. Direct measurement corroborated that the ATP concentration was not altered (Figure 3h). Hence, absence of Lsd1 activity for 24 h induces mitochondrial stress indicated by decreased basal oxygen consumption, increased mitochondrial membrane potential and glycolysis. In accordance with our metabolomics data, this could be caused by a reduced substrate supply from glutamine anaplerosis.

Increased membrane potential with curtailed oxygen consumption suggests increased ROS levels. Accordingly, we quantified H_2_O_2_ in the cytoplasm and in mitochondria using ratiometric roGFP2-based redox probes.^48^ Inhibition of Lsd1 for 24 h induced a raise in H_2_O_2_ in both cellular compartments (Figure 3i). Our data were confirmed by measurements of overall ROS formation. Using DCF as a dye that becomes fluorescent upon oxidation, we observed an increase in relative signal intensity upon Lsd1 inhibition (Figure 3j). We also evaluated the level of glutathione, the major redox reagent in the cytoplasm and in mitochondria, using ratiometric roGFP2-based redox reporters.^48^ Inhibition of Lsd1 for 24 h raised the relative amount of oxidized glutathione (GSSG) in both compartments (Figure 3k). Consistently, reducing power was diminished as indicated by the increased ratio of oxidized to reduced levels of NADP, NAD and decreased GSH concentration (Figures 3l and m). In brief, short-term inhibition of Lsd1 raises ROS-mediated stress, which is exacerbated by diminished reducing capacity. The latter may be based on the decreased glutamine anaplerosis leading to a decline in GSH synthesis.

We next investigated the impact of Lsd1 on mitochondrial function at a later time point 8 days after the induction of Lsd1 deletion. Similar to short-term Lsd1 inhibition, the basal OCR was reduced in *Lsd1*^−/−^ TSCs. In contrast, reserve capacity, mitochondrial membrane potential, and ATP levels were reduced displaying that the initial mitochondrial stress caused by the absence of Lsd1 leads to mitochondrial damage over time (Supplementary Figures S2c–e). H_2_O_2_ and ROS were maintained at an elevated level and the amount of reducing metabolites remained low in *Lsd1*-deficient TSCs (Supplementary Figures S2f–j). In summary, loss of Lsd1 diminishes glutamine anaplerosis essential to maintain redox balance and functional mitochondria.

### Sirt4 triggers senescence in TSCs

**Figure 4:**
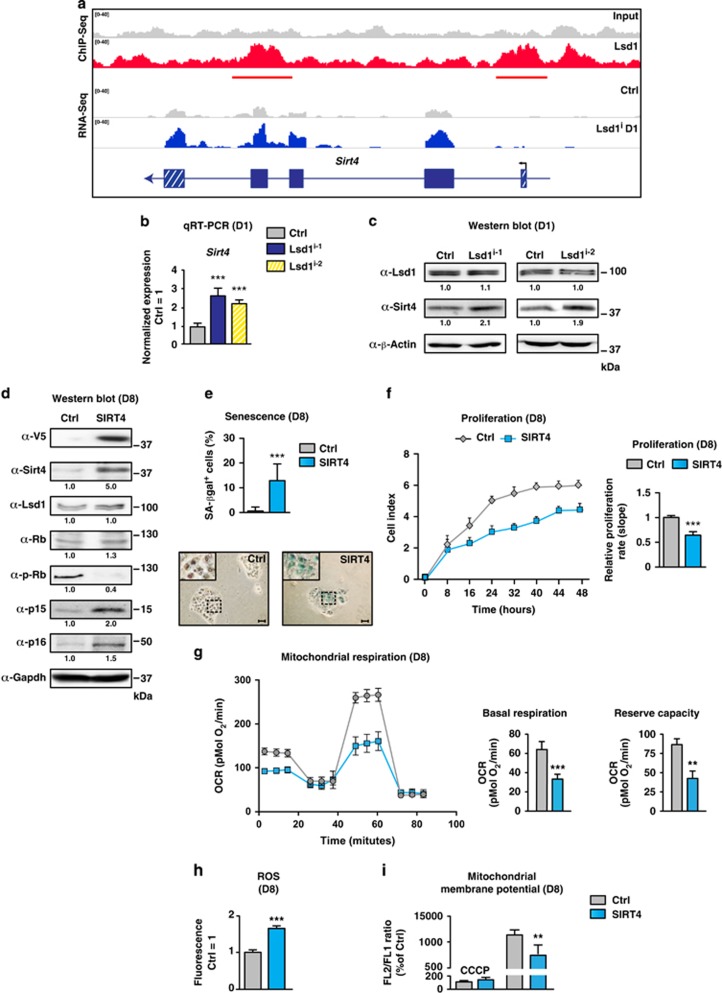
Sirt4 triggers senescence in TSCs. (**a**–**c**) Comparison of TSCs cultured for 24 h in the presence of solvent (Ctrl), Lsd1 inhibitor 1 (Lsd1^i-1^) (**a**–**c**) and Lsd1 inhibitor 2 (Lsd1^i-2^) (**b** and **c**). (**a**) Localization of Lsd1 at the *Sirt4* gene and differential *Sirt4* transcription. (**b** and **c**) Quantification of Sirt4 expression by qRT-PCR (**b**) and western blot (**c**). Data were normalized to three housekeeping genes (**b**) and *β*-actin as loading control relative to the Ctrl (**c**). (**d**–**i**) Comparison of TSCs transfected with an empty vector (Ctrl) or SIRT4 expression plasmid. (**d**) Western blot for the indicated antibodies including V5-tagged SIRT4 and phosphorylated Rb (p-Rb). Band intensity was normalized to Gapdh relative to the Ctrl. (**e**) Quantification of senescent cells and representative images of senescent-associated *β*-galactosidase activity (blue) in TSCs. (**f**) Proliferation of TSCs was determined in real time. (**g**) Mitochondrial respiration was determined by a time course of OCR. Complex V was blocked by oligomycin (O), uncoupling was induced by FCCP (F) and electron transport system was disabled by addition of rotenone (R) and antimycin A (A). Basal respiration is the difference of the OCR in the absence of inhibitors and after addition of rotenone and antimycin A. The reserve capacity is derived from the subtraction of the OCR after addition of rotenone and antimycin A from oligomycin-treated TSCs. (**h**) Relative quantification of ROS using fluorescent dye. (**i**) Relative mitochondrial membrane potential determined by FL2/FL1 ratio. The background level was assigned by depolarization with CCCP. Data were analyzed from at least three biological samples and are represented as mean+S.E.M. **P<0.01; ***P<0.001 (unpaired, two-tailed Student's *t*-test, (**b**, **e** and **f**–**i**))

To uncover the immediate early molecular mechanism triggering senescence in TSCs, we screened differentially expressed direct targets of Lsd1 involved in metabolic pathways, regulation of mitochondrial activity and glutamine anaplerosis. We considered *Sirt4* as the most promising candidate. *Sirt4,* a direct target of Lsd1 and upregulated by inhibition of Lsd1 (Figure 4a), is known to restrain proton leakage^21^ and glutaminolysis by repression of glutamine uptake and Glud1 activity.^22, 23, 49^ We validated the increase in Sirt4 expression after inhibition of Lsd1 by qRT-PCR (Figure 4b) and by western blot (Figure 4c). Consequently, we were keen to link the impact of enhanced *Sirt4* expression in wild-type TSCs to senescence, glutamine anaplerosis, redox balance and mitochondrial function. Transient transfection of a *SIRT4* expression plasmid raised the levels of cell cycle inhibitors p15, p16 and Rb without altering transcription of endogenous Lsd1 and Sirt4 (Figure 4d and Supplementary Figure S3a). Accordingly, the number of senescence-associated *β*-galactosidase-positive cells was increased (Figure 4e) and proliferation was curtailed (Figure 4f) similar to the phenotype observed in TSCs cultured in the presence of Lsd1 inhibitor for 8 days (Supplementary Figures S1a and b). In comparison with mock-transfected TSCs, glutamine uptake (Supplementary Figure S3b) and Glud1 activity (Supplementary Figure S3c) were reduced in SIRT4-overexpressing cells indicating that glutamine anaplerosis is decreased by Sirt4 in TSCs. Similarly, the levels of ammonia and GSH were diminished upon enhanced expression of *SIRT4* in comparison with control cells (Supplementary Figures S3d and e). Next, we determined mitochondrial oxygen consumption. TSCs transfected with *SIRT4* expression vector exhibited a phenotype comparable to *Lsd1*-deficient TSCs. Two and 8 days after transfection, basal oxygen consumption and reserve capacity were diminished (Figure 4g and Supplementary Figure 3f) with increased ROS levels (Figure 4h and Supplementary Figure S3g). The dynamic of the mitochondrial phenotype in the absence of Lsd1 activity was recapitulated by Sirt4 overexpression. The mitochondrial membrane potential was initially increased 48 h after transfection while it was decreased after 8 days (Supplementary Figure S3h and Figure 4i).

To analyze the impact of decreased glutamine anaplerosis on senescence in TSCs, we inhibited glutaminase (Gls), which converts glutamine to glutamate, by BPTES^50^ or compound 968.^51^ Oxidation of glutamate to *α*-ketoglutarate by Glud1 was inhibited by EGCG.^52^ Either inhibition enhanced the expression of cell cycle inhibitors p16 and p15 and Rb while the transcription of *Lsd1* was not affected (Supplementary Figure S3i). Furthermore, senescence-associated *β*-galactosidase activity was increased (Supplementary Figure S3j). Thus, direct inhibition of glutamine anaplerosis triggers senescence in TSCs similar to SIRT4 overexpression or Lsd1 inhibition.

### Knockdown of Sirt4 prevents redox imbalance in the presence of Lsd1 inhibitor

**Figure 5:**
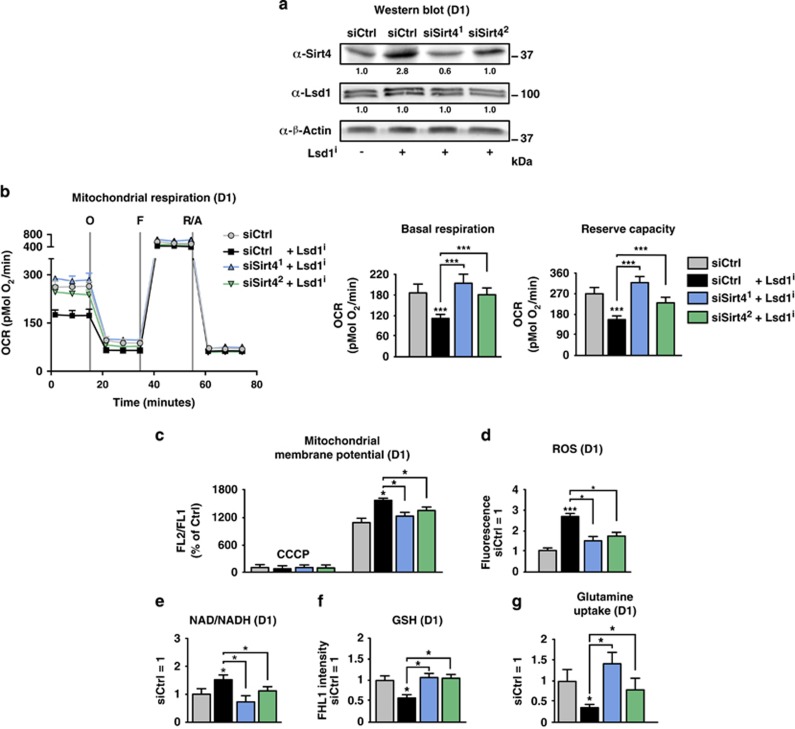
Knockdown of Sirt4 prevents redox imbalance in the presence of Lsd1 inhibitor. (**a**–**g**) Comparison of TSCs cultured in the presence of Lsd1 inhibitor 1 (Lsd1^i-1^) or solvent and transfected with an unrelated or two different siRNAs directed against Sirt4. (**a**) Western blot decorated with the indicated antibodies. Band intensity was normalized to *β*-actin relative to the Ctrl transfected with an unrelated siRNA in the absence of Lsd1 inhibitor. (**b**) Mitochondrial respiration was determined by a time course of OCR. Complex V was blocked by oligomycin (O), uncoupling was induced by FCCP (F) and electron transport system was disabled by addition of rotenone (R) and antimycin A (A). Basal respiration is the difference of the OCR in the absence of inhibitors and after addition of rotenone and antimycin A. The reserve capacity is derived from the subtraction of the OCR after addition of rotenone and antimycin A from oligomycin-treated TSCs. (**c**) Relative mitochondrial membrane potential was determined by FL2/FL1 ratio. The background level was assigned by depolarization with CCCP. (**d**) Relative quantification of ROS using fluorescent dye. (**e**–**g**) Relative ratio of oxidized to reduced NADP and NAD (**e**), glutathione levels (**f**) and glutamine uptake (**g**). Data were analyzed from at least three biological samples and are represented as mean+S.E.M. *P<0.05, ***P<0.001 (unpaired, two-tailed Student's *t*-test, (**b**–**g**))

To prevent the imbalance of the redox potential triggering senescence in TSCs devoid of Lsd1 demethylase activity, we reduced Sirt4 by RNAi. Reduction of endogenous Sirt4 expression by two specific siRNAs in the presence of Lsd1 inhibitor was verified by western blot (Figure 5a). We first analyzed mitochondrial function. Inhibition of Lsd1 in TSCs transfected with an unrelated control siRNA decreased the basal OCR. In contrast, knockdown of Sirt4 by two independent siRNAs returned the basal OCR to the level of control cells (Figure 5b). Similarly, reserve capacity and membrane potential were restored to control levels (Figures 5b and c). Similarly, ROS caused by the inhibition of Lsd1 was curtailed by simultaneous transfection of siRNAs directed against Sirt4 (Figure 5d). Accordingly, the relative levels of NADH and GSH were elevated upon Sirt4 reduction despite the presence of Lsd1 inhibitor (Figures 5e and f). Similarly, glutamine uptake was augmented by knockdown of Sirt4 in the presence of Lsd1 inhibitor (Figure 5g). Taken together, the imbalance of the redox potential caused by inhibition of Lsd1 can be averted by concomitant reduction of Sirt4 expression.

### Lsd1 controls senescence by repression of Sirt4

**Figure 6:**
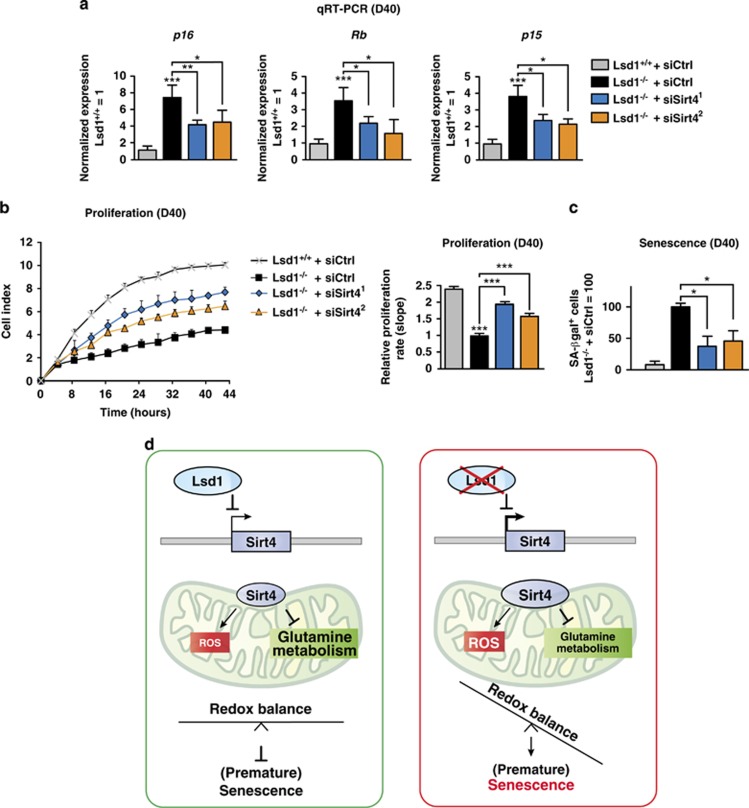
Lsd1 controls Senescence by Repression of *Sirt4*. (**a**–**c**) Comparison of wild-type (*Lsd1*^+/+^) and *Lsd1*-deficient (*Lsd1*^−/−^) TSCs transfected with an unrelated or two different siRNAs directed against Sirt4. (**a**) qRT-PCR of the indicated cell cycle regulators normalized to three housekeeping genes relative to transcription in wild-type TSCs transfected with an unrelated siRNA. (**b**) Proliferation of TSCs was determined in real time. (**c**) Quantitative determination of senescent TSCs. (**d**) Model for the Lsd1-controlled redox balance preventing senescence. Data were analyzed from at least three biological samples and are represented as mean+S.E.M. *P<0.05, ***P<0.001 (unpaired, two-tailed Student's *t*-test, (**a**–**c**))

To finally prove that Lsd1 prevents senescence by repression of *Sirt4* in TSCs, we reduced Sirt4 expression in *Lsd1*^−/−^ TSCs. Specific knockdown of Sirt4 was verified by western blot (Supplementary Figure S4a). Expression of the cell cycle inhibitors p15 and p16 and Rb were decreased and phosphorylation of Rb increased (Figure 6a and Supplementary Figure S4a). Accordingly, proliferation was enhanced in *Lsd1*^−/−^ TSCs upon Sirt4 knockdown (Figure 6b). Importantly, knockdown of *Sirt4* in *Lsd1*-deficient TSCs markedly decreased the percentage of senescence-associated *β*-galactosidase-positive cells in comparison with the control (Figure 6c and Supplementary Figure S4b).

In summary, we demonstrate that *Lsd1* deletion triggers senescence in TSCs, by unravelling a key role of Lsd1 to sustain metabolic flexibility. We propose the following model (Figure 6d). Lsd1 binds to the promoter of *Sirt4* and impedes transcription. Consequently, glutamine anaplerosis and GSH synthesis are not restrained by *Sirt4* expression. In parallel, enhanced mitochondrial membrane potential exacerbated by diminished oxygen consumption favouring ROS is averted. Thus, the redox level is kept in balance preventing stress-induced senescence.

## Discussion

Our data show that the demethylase activity of Lsd1 governs redox homeostasis and metabolic flexibility to control senescence in TSCs. The increase in *Sirt4* transcription after Lsd1 inhibition in conjunction with binding of Lsd1 to the *Sirt4* gene locus indicates that the expression of *Sirt4* is directly repressed by Lsd1. Overexpression of Sirt4 in TSCs is sufficient to increase the mitochondrial membrane potential accompanied by simultaneous reduction of oxygen consumption, a combination, which favours the emergence of ROS. We suggest that loss of redox and metabolic balance triggers senescence in TSCs devoid of functional Lsd1 (Figure 6d).

The raise in Sirt4 expression in TSCs devoid of Lsd1 activity interferes with glutamine anaplerosis. Surprisingly though, we also do observe increased levels of fructose 6-phosphate, 3-phosphoglyceric acid, pyruvate and citrate after inhibition of Lsd1 proposing an enhanced glycolytic catabolism into the TCA cycle. This observation is counterintuitive, because Sirt4 has been associated with the inhibition of the pyruvate dehydrogenase complex.^53^ The effect of Lsd1 inhibition on glucose metabolism is further complicated by decreased levels of serine, glycine and cysteine, which all can be derived from 3-phosphoglycerate. The low levels of these amino acids may be due to the importance of glutamine anaplerosis as the main nitrogen donor for the synthesis of amino acids ^54^ or to an increased glycolytic flux.^55^ Furthermore, we noticed decreased cysteine levels suggesting that the one-carbon cycle is diminished. This is corroborated by a 70% reduction of the methyl donor *S*-adenosyl methionine. In the future, it will be interesting to further analyze the impact of the histone demethylase Lsd1 on the substrate level for histone and DNA methyltransferases. Lsd1 may direct a potential feedback mechanism, linking metabolism with chromatin methylation.

In the context of senescence triggered by Lsd1 inhibition, it is compelling to note that LSD1 and SIRT4 control proliferation in cancer cells. Enhanced LSD1 expression has been linked to tumour progression^56, 57, 58, 59^ and can even prime cells for transformation.^60^ Consequently, clinical trials are currently ongoing to implement LSD1 inhibitors for the treatment of refractory small cell lung cancer (NCT02034123) and acute myeloid leukaemia (NCT02177812, NCT02273102 and NCT02261779). Inversely, SIRT4 can function as a tumour suppressor and reduced expression is associated with several cancers.^22, 61^ Furthermore, *Sirt4*-deficient mice spontaneously develop lung tumours.^24^ Many cancer cells heavily depend on glutaminolysis to couple bioenergetics to redox balance, supporting cell survival.^54, 62^ The LSD1-directed control of glutamine anaplerosis offers an explanation for the abatement on tumour progression by LSD1 inhibitors in cancers. Excitingly, our data suggest that Lsd1 inhibition also causes increased glycolytic flux at least in TSCs. In the future it will be interesting to analyze the impact of LSD1 on glucose anaplerosis in other stem and cancer (stem) cells. Treatment with LSD1 inhibitors and simultaneous repression of glycolysis may enhance the efficacy of treatment by killing senescent cancer cells but also could increase the range of tumours susceptible to growth inhibition by LSD1 inhibitors.

Of note, inhibition of Lsd1 has never been connected with senescence, indicating that the cellular context is important for the control of proliferation by Lsd1. Unravelling the corresponding molecular mechanism and identification of responsive cell types may pave the way for an extension of cancers susceptible to co-treatment with Lsd1 inhibitors and compounds affecting central metabolic pathways. Stem cell maintenance during aging and obliteration of cancer stem cells during tumour progression are connected by chromatin modifications and the control of metabolism. Thus, the histone demethylase Lsd1 is a very attractive target integrating the control of chromatin and metabolism.

## Materials and Methods

### TSC culture

For clarity, TSCs homozygous for a conditional *Lsd1* allele are defined as wild-type (*Lsd1*^+/+^). The clonal cell lines are derived from two different embryos. Recombination of the conditional *Lsd1* alleles was induced with 0.2 *μ*M 4OH-tamoxifen (Sigma, Munich, Germany, H-7904) 8 days before the start of the experiment. Recombination causes deletion of exon 1 (*Lsd1*^−/−^) or replacement of exon 15 to 19 with a triple point mutation encoding K662A, W752A and Y762S (*Lsd1*^ki/ki^). For inhibition, TSCs were cultured 24 h or 8 days before and during the experiment in the presence of 100 nM Lsd1 inhibitor 1 (ORY-1001, Selleckchem, Munich, Germany),^43^ 100 nM Lsd1 inhibitor 2 (QC6688), 50 *μ*M EGCG (Sigma, 50299) 10 *μ*M BPTES (Sigma, SM0601), 20 *μ*M 968 (Millipore, Darmstadt, Germany, 352010) or solvent (DMSO, Sigma, C6295). TSCs were transfected in suspension after culturing for two passages on feeders according to Tanaka *et al.*^63^ using lipofectamine LTX (Thermo Fisher Scientific, Schwerte, Germany, 15338100) for plasmids or Lipofectamine RNAimax (Thermo Fisher Scientific, 13778030) for siRNA. TSCs were transfected with stealth siRNA directed against mouse Sirt4 or an unrelated control (Supplementary Table S2) 48 h before start of treatment with Lsd1 inhibitor or 4OH-tamoxifen and maintained for the duration of the experiment. TSCs transfected with pEF-DEST52-Cherry-SIRT4 or empty vector were selected by FACS. During selection and duration of the experiment, the cells were incubated in stemness conditions (cell culture medium containing 25 ng/ml human recombinant FGF4 (Cell Concepts, Umkirch, Germany, C-10312M), 1 *μ*g/ml Heparin (Sigma, H3149) and 75% feeder-conditioned medium).

### Proliferation

Assays were performed using xCelligence system (OLS-OMNI Life Science, Bremen, Germany) system by seeding 1 × 10^5^ cells per well in E-8 chambers (OLS-OMNI Life Science) in stemness medium. Cell indices were automatically recorded every 15 min. Relative proliferation velocity was calculated by using the slope.

### Senescence-associated *β*-galactosidase activity analyses

*In situ* analysis of *β*-galactosidase activity was performed as described in Debacq-Chainiaux *et al.*^64^ Briefly, subconfluent cells were fixed with 2% paraformaldehyde (PFA; Sigma, P6148), 0.2% glutaraldehyde (Sigma, G5882) in phosphate-buffered saline (PBS), washed with PBS and stained with solution containing 1 mg ml-1 X-gal (Sigma, B4252). For quantification of senescent cells, TSCs were stained with C12FDG (Thermo Fisher Scientific, D2893) and analyzed by FACS.

### Western blot analysis and immunofluorescence

Whole-cell lysate was extracted from TSCs as described.^30^ Briefly, cells were lysed in SC buffer and protein extract supernatants were resolved using acrylamide gels and electrotransfered to PVDF membranes (Millipore, IPVH00010), according to the manufacturer's instructions. Membranes were incubated in blocking buffer for 1 h at room temperature, decorated with antibodies directed against Lsd1 (Schüle laboratory, Freiburg, Germany, 3544 (ref. 30) 1:1000), Rb (Santa Cruz, Heidelberg, Germany, sc-50, 1:200), p-Rb (Cell Signaling, Frankfurt, Germany, 2181, 1:500), p15 (Abcam, Cambridge, UK, ab53034, 1:500), p16 (Santa Cruz, sc-1661, 1:200), Gapdh (Millipore, MAB374, 1:300), Sirt4 (Abcam, ab10140, 1:100) and *β*-Actin (Sigma, A1978, 1:2000) p21 (Santa Cruz, sc-6246, 1:500), p53 (Cell Signaling, 9282, 1:1000), phospho-p53 (Cell Signaling, 9286, 1:1000), histone H2ax (Abcam, ab07–627, 1:2000), phospho histone H2ax (Millipore, 05–636, 1:1000) and histone H3 (Abcam, ab1791, 1:5000) in blocking buffer at 4 °C overnight. Blots were washed with 0.1% PBST and incubated with HRP-conjugated secondary antibodies. Chemiluminescence was detected and images were acquired with a CHEMISMART documentation system and the Chemi-Capt 5000 software (Peqlab, Vilber Lourmat, Eberhardzell, Germany) and Image Quant TL software (General Electric, Munich, Germany). Quantitative analysis for normalization was performed with the Bio-1D software (Vilber Lourmat). The signal was normalized to the internal loading control and expressed relative to the sample control.

Immunofluorescence analysis was performed by fixation with 4% PFA for 10 min followed by three wash with PBS, permeabilization in 0.2% TritonX-100/PBS for 15 min, wash and overnight blocking with 0.2% gelatin the cells were then incubated with a primary Lsd1 antibody (Sigma, 112-4, 1 : 500). After washing, cells were incubated with the correspondent secondary antibodies (Thermo Fisher Scientific, *α*-rabbit Alexa Fluor 488) and 4',6-diamidino-2-phenylindole (DAPI) (Thermo Fisher Scientific, 62248, 1:10000).

### RNA extraction, qRT-PCR and RNA-seq

RNA was isolated with Trizol (Thermo Fisher Scientific, 15596), reverse transcription and qPCR were performed as described.^30^ Transcription of *Ppia, Rplp0* and *Hprt* was used for normalization and expression was related to the control sample. Experiments were repeated at least with three biological samples. Primers are listed in Supplementary Table S2.

RNA-seq analysis was performed according to standard Illumina protocol. Raw sequence files (.fastq files) were aligned to mouse genome (mm10) using TopHat version 2.^65^ Counting of the aligned reads was performed using HOMER software (analyzeRNA.pl). Differentially expressed genes were identified with EdgeR software.^66^

### ChIP-seq

TSCs cultured in stemness conditions were fixed with 1% PFA in 20 mM Tris-HCL pH 8, 0.5 mM EGTA, 1 mM EDTA, 10 mM NaCl for 5 min, quenched with glycine for 5 min and rinsed twice with ice-cold PBS. Cells were harvested in PBS supplemented with Complete EDTA-free protease inhibitors cocktail (Roche, Mannheim, Germany, 11873580001) pelleted and flash-frozen in liquid nitrogen. Chromatin preparation was done as previously described.^67^ Fixed cells were resuspended in Farnham Lab buffer and nuclei were extracted using an ultrasound-based nuclei extraction method. Nuclei were pelleted, resuspended in shearing buffer and sonicated for 15 min using Covaris S220 (Brighton, UK, instrument settings: 140 W peak power, 5% duty factor, 200 cycles/burst, at 4 °C), to a fragment size distribution of 100–800 bp.

ChIP was performed using the automated platform IP-Star SX-8G Compact (Diagenode, Oxford, UK) and Auto Histone ChIP-seq kit (Diagenode, C01010022) following the ‘indirect method' procedure. ChIP reactions were incubated with 2 *μ*g Lsd1 antibody 20752 (ref. 40) for 10 h, followed by 3 h of incubation with beads and 5-min washes at 4 °C. Libraries were prepared from immunoprecipitated DNA using NEBNextUltra (Frankfurt, Germany) library preparation kit without size selection. ChIP-seq libraries were sequenced using a HiSeq 2500 (Illumina, Cambridge, UK) and mapped to the mm10 reference genome using bowtie 2 (ref. 68). Data were further analyzed using the peak finding algorithm MACS 1.41 (ref. 69) using input as control. All peaks with FDR >1% were excluded from further analysis. The uniquely mapped reads were used to generate the genome-wide intensity profiles, which were visualized using the IGV genome browser.^70^ HOMER ^71^ was used to annotate peaks. The genomic features were defined and calculated using Refseq and HOMER.

### Metabolite analyses

TSCs were snap frozen in liquid nitrogen and stored at −80 °C. Sample preparation was performed according to metaSysX standard procedure, a modified protocol from Giavalisco *et al.*^72^ Sample processing and measurement were performed with an ACQUITY RP-UPLC (Waters, Eschborn, Germany) coupled to an Exactive mass spectrometer (Thermo Fisher Scientific) GC-MS measurements were performed on a GC (Agilent Technologies, Waldbronn, Germany) coupled to a Pegasus HT mass spectrometer (Leco, Saint Joseph, MI, USA). UPLC-MS and GC-MS measurements were performed for the analysis of primary and secondary metabolites. After extraction from the chromatograms, the data were processed, aligned, filtered and annotated according to metaSysX standard procedures.

### Oxygen consumption and extracellular acidification rate

Mitochondrial OCRs were analyzed using XFp analyzer^73^ (Seahorse Bioscience, Waldbronn, Germany) according to the manufacturer's instructions in RPMI medium without glucose and sodium bicarbonate (R1383, Sigma), supplemented with 10 mM glucose (G8644, Sigma) and 1 mM pyruvate (P5280, Sigma) using 2 *μ*M oligomycin, 0.6 *μ*M rotenone/antimycin and 0.7 *μ*M FCCP (XFp cell mito stress test kit, Seahorse Bioscience). In all, 5 × 10^4^ TSCs were seeded 24 h before the beginning of the assay. Data were normalized to protein content after the assay.

Mitochondrial membrane potential (JC1 assay) was assessed using MitoProbe JC-1 Assay Kit for Flow Cytometry (Thermo Fisher Scientific) following the manufacturer's instructions. Briefly, TSCs were cultured in the presence of the mitoprobe 2 *μ*M 5',6,6'-tetrachloro-1,1',3,3'-tetraethylbenzimidazolylcarbocyanine iodide (JC-1). Background measurements were obtained using 50 *μ*M CCCP (carbonyl cyanide 3-chlorophenylhydrazone). JC-1 fluorescent ratio from 590/529 nm (Fl2/FL1 channels) was calculated to estimate the relative membrane potential before and after the indicated treatment.

### ROS and redox quantification

TSCs under the indicated treatment were transfected with ratiometric redox plasmids pLPCX-Grx1-roGFP2 (used for cytoplasmatic GSH), pLPCX-mito-Grx1-roGFP2 (used for mitochondrial GSH), pLPCX-roGFP2-Orp1 (used for cytoplasmatic H_2_O_2_) and pLPCX-mito-roGFP2-Orp1(used for mitochondrial H_2_O_2_) (ref. 48) and analyzed by FACS. The 405/488 nm ratio was calculated for gated GFP-positive cells.

Cellular ROS content was measured by incubation with 5 *μ*M H2DCF-DA (Thermo Fisher Scientific) for 20 min in phenol red-free medium. The cells were washed in PBS buffer, trypsinized and resuspended in 500 *μ*l PBS.

Total glutathione (GSH) levels were tested using intracellular GSH assay kit (Abcam) following the manufacturer's instructions. Fluorescence intensity was visualized by flow cytometry using FL1 channel.

### FACS

FACS was performed with Aria or Fortessa flow cytometer (BD Biosciences, Heidelberg, Germany) by recording 10 000 events. Data were analyzed using the Flow Jo (Ashland OR, USA) 7.5.5 and FACSDiva software (Heidelberg, Germany). For quantification of cell cycle inhibitors, TSCs were harvested with 0.05% trypsin/EDTA, resuspended in FACS buffer (2% FBS in PBS) and filtered through a 40 *μ*m cell strainer, fixed with 4% PFA 10 min at RT. After fixation cells were permeabilized in FACS buffer 0.1% Tween 20 (Sigma, P7949) for 30 min and blocked with 10% FBS overnight. Blocked cells were resuspended in FACS buffer with Cdkn2a Alexa fluor 488 (Abcam, ab199756; 1:50), rabbit IgG isotype control Alexa fluor 488 (Cell Signaling, 4340s, 1:100) or phospho-Rb (Cell Signaling, 4277, 1:50) antibody, and incubated overnight at 4 °C. After washing, cells were resuspended in FACS buffer for acquisition.

### Biochemical assays

Quantification of NAD and NADP was performed according to the manufacturer's instructions (Abcam, ab65348) and (Abcam, ab65349), respectively. Briefly, 2 × 10^6^ cells were suspended in respective assay extraction buffer by freeze/thaw cycles. The cells supernatants were deproteinized in 10 kD spin columns. NADPH/NADH levels were measured in the resulting samples by NADP/NAD heat decomposition (60 °C for 30 min).

Ammonia content was measured using Assay kit (Abcam, ab83360), according to the manufacturer's instructions. Briefly, 2 × 10^6^ cells were homogenized in assay buffer, after centrifugation the supernatants were diluted to fit standard curve and resolved in a microplate reader. Pyruvate control levels were subtracted for each sample.

ATP levels were measured using Assay kit (Abcam, ab83355), according to the manufacturer's instructions. Briefly, 2 × 10^6^ cells were suspended by pipetting up and down in assay buffer. The samples were deproteinized by PCA precipitation and diluted at different concentrations to ensure reading within the standard curve and resolved by fluorometric assay.

Glutamate dehydrogenase activity was measured according to the manufacturer's instructions (Sigma, MAK099). Briefly, 1 × 10^6^ cells were homogenized in assay buffer. After 10 min of incubation, the insoluble material was removed by centrifugation. The samples were measured by colorimetric assay and the Glud1 activity calculated by the ratio difference between the initial and final 450 nm absorbance.

For quantification of glutamine uptake, 1 × 10^6^ TSCs were seeded. The medium was replaced and collected after 24 h, centrifuged and the glutamine levels in the supernatant were measured according to the manufacturer's instructions (Biovision, Wehrheim, Germany, K556).

### Plasmids

Expression plasmids for SIRT4 were generated by BP and LR recombination according to the supplier (Thermo Fisher Scientific). The expression plasmid pDest26-SIRT4 (Source Bioscience, Berlin, Germany, IOHx59335) was used for the generation of a zeocin-resistant entry clone and expression plasmids containing mCherry or CFP reporters. The QuickChange II site-directed mutagenesis kit (Agilent Technologies) was used to delete the translational STOP codon of SIRT4 to generate a V5-tagged fusion protein. The redox potential was quantified by transfection of pLCPX-mito-Grx1-roGFP2, pLCPX-Grx1-roGFP2 (ref. 48). For determination of intracellular hydrogen peroxide concentration pLCPX-mito-Orp1-roGFP2 and pLCPX-mito-Orp1-roGFP2 were used.^48^

### Statistical analysis

If not otherwise stated, significance was calculated using an unpaired, two-tailed Student's *t*-test. Data are calculated as mean +S.E.M. Experiments were repeated at least three times. Principal component and cluster analysis were used as descriptive methods to confirm or reject discrimination between the sample groups. Overrepresentation of candidate genes in regards to classification was determined by binominal test.

### Accession codes

RNA-Seq and ChIP-seq data have been deposited in Gene Expression Omnibus (GEO) data base under accession codes GSE78782 and GSE78264, respectively.

## References

[bib1] Oh J, Lee YD, Wagers AJ. Stem cell aging: mechanisms, regulators and therapeutic opportunities. Nat Med 2014; 20: 870–880. 25100532 10.1038/nm.3651 PMC4160113

[bib2] Bratic A, Larsson NG. The role of mitochondria in aging. J Clin Invest 2013; 123: 951–957. 23454757 10.1172/JCI64125 PMC3582127

[bib3] Hayflick L, Moorhead PS. The serial cultivation of human diploid cell strains. Exp Cell Res 1961; 25: 585–621. 13905658 10.1016/0014-4827(61)90192-6

[bib4] Kawahara TL, Michishita E, Adler AS, Damian M, Berber E, Lin M et al. SIRT6 links histone H3 lysine 9 deacetylation to NF-kappaB-dependent gene expression and organismal life span. Cell 2009; 136: 62–74. 19135889 10.1016/j.cell.2008.10.052 PMC2757125

[bib5] Lopez-Otin C, Blasco MA, Partridge L, Serrano M, Kroemer G. The hallmarks of aging. Cell 2013; 153: 1194–1217. 23746838 10.1016/j.cell.2013.05.039 PMC3836174

[bib6] Munoz-Espin D, Serrano M. Cellular senescence: from physiology to pathology. Nat Rev Mol Cell Biol 2014; 15: 482–496. 24954210 10.1038/nrm3823

[bib7] Serrano M, Lin AW, McCurrach ME, Beach D, Lowe SW. Oncogenic ras provokes premature cell senescence associated with accumulation of p53 and p16INK4a. Cell 1997; 88: 593–602. 9054499 10.1016/s0092-8674(00)81902-9

[bib8] Dimri GP, Lee X, Basile G, Acosta M, Scott G, Roskelley C et al. A biomarker that identifies senescent human cells in culture and in aging skin *in vivo*. Proc Natl Acad Sci USA 1995; 92: 9363–9367. 7568133 10.1073/pnas.92.20.9363 PMC40985

[bib9] Narita M, Nunez S, Heard E, Narita M, Lin AW, Hearn SA et al. Rb-mediated heterochromatin formation and silencing of E2F target genes during cellular senescence. Cell 2003; 113: 703–716. 12809602 10.1016/s0092-8674(03)00401-x

[bib10] Gorrini C, Harris IS, Mak TW. Modulation of oxidative stress as an anticancer strategy. Nat Rev Drug Discov 2013; 12: 931–947. 24287781 10.1038/nrd4002

[bib11] Bigarella CL, Liang R, Ghaffari S. Stem cells and the impact of ROS signaling. Development 2014; 141: 4206–4218. 25371358 10.1242/dev.107086 PMC4302918

[bib12] Harman D. Aging: a theory based on free radical and radiation chemistry. J Gerontol 1956; 11: 298–300. 13332224 10.1093/geronj/11.3.298

[bib13] Ito K, Hirao A, Arai F, Matsuoka S, Takubo K, Hamaguchi I et al. Regulation of oxidative stress by ATM is required for self-renewal of haematopoietic stem cells. Nature 2004; 431: 997–1002. 15496926 10.1038/nature02989

[bib14] Liu H, Wang H, Shenvi S, Hagen TM, Liu RM. Glutathione metabolism during aging and in Alzheimer disease. Ann N Y Acad Sci 2004; 1019: 346–349. 15247041 10.1196/annals.1297.059

[bib15] Lu SC. Regulation of glutathione synthesis. Mol Aspects Med 2009; 30: 42–59. 18601945 10.1016/j.mam.2008.05.005 PMC2704241

[bib16] Wang H, Liu H, Liu RM. Gender difference in glutathione metabolism during aging in mice. Exp Gerontol 2003; 38: 507–517. 12742528 10.1016/s0531-5565(03)00036-6

[bib17] Orlowski M, Meister A. The gamma-glutamyl cycle: a possible transport system for amino acids. Proc Natl Acad Sci USA 1970; 67: 1248–1255. 5274454 10.1073/pnas.67.3.1248 PMC283344

[bib18] Houtkooper RH, Pirinen E, Auwerx J. Sirtuins as regulators of metabolism and healthspan. Nat Rev Mol Cell Biol 2012; 13: 225–238. 22395773 10.1038/nrm3293 PMC4872805

[bib19] Zhong L, Mostoslavsky R. Fine tuning our cellular factories: sirtuins in mitochondrial biology. Cell Metab 2011; 13: 621–626. 21641544 10.1016/j.cmet.2011.05.004 PMC3111451

[bib20] Nakamura Y, Ogura M, Tanaka D, Inagaki N. Localization of mouse mitochondrial SIRT proteins: shift of SIRT3 to nucleus by co-expression with SIRT5. Biochem Biophys Res Commun 2008; 366: 174–179. 18054327 10.1016/j.bbrc.2007.11.122

[bib21] Ho L, Titus AS, Banerjee KK, George S, Lin W, Deota S et al. SIRT4 regulates ATP homeostasis and mediates a retrograde signaling via AMPK. Aging (Albany, NY) 2013; 5: 835–849. 24296486 10.18632/aging.100616 PMC3868726

[bib22] Csibi A, Fendt SM, Li C, Poulogiannis G, Choo AY, Chapski DJ et al. The mTORC1 pathway stimulates glutamine metabolism and cell proliferation by repressing SIRT4. Cell 2013; 153: 840–854. 23663782 10.1016/j.cell.2013.04.023 PMC3684628

[bib23] Haigis MC, Mostoslavsky R, Haigis KM, Fahie K, Christodoulou DC, Murphy AJ et al. SIRT4 inhibits glutamate dehydrogenase and opposes the effects of calorie restriction in pancreatic beta cells. Cell 2006; 126: 941–954. 16959573 10.1016/j.cell.2006.06.057

[bib24] Jeong SM, Xiao C, Finley LW, Lahusen T, Souza AL, Pierce K et al. SIRT4 has tumor-suppressive activity and regulates the cellular metabolic response to DNA damage by inhibiting mitochondrial glutamine metabolism. Cancer Cell 2013; 23: 450–463. 23562301 10.1016/j.ccr.2013.02.024 PMC3650305

[bib25] Gut P, Verdin E. The nexus of chromatin regulation and intermediary metabolism. Nature 2013; 502: 489–498. 24153302 10.1038/nature12752

[bib26] Ryall JG, Cliff T, Dalton S, Sartorelli V. Metabolic reprogramming of stem cell epigenetics. Cell Stem Cell 2015; 17: 651–662. 26637942 10.1016/j.stem.2015.11.012 PMC4672395

[bib27] Shi Y, Lan F, Matson C, Mulligan P, Whetstine JR, Cole PA et al. Histone demethylation mediated by the nuclear amine oxidase homolog LSD1. Cell 2004; 119: 941–953. 15620353 10.1016/j.cell.2004.12.012

[bib28] Shi YJ, Matson C, Lan F, Iwase S, Baba T, Shi Y. Regulation of LSD1 histone demethylase activity by its associated factors. Mol Cell 2005; 19: 857–864. 16140033 10.1016/j.molcel.2005.08.027

[bib29] Metzger E, Imhof A, Patel D, Kahl P, Hoffmeyer K, Friedrichs N et al. Phosphorylation of histone H3T6 by PKCbeta(I) controls demethylation at histone H3K4. Nature 2010; 464: 792–796. 20228790 10.1038/nature08839

[bib30] Metzger E, Wissmann M, Yin N, Müller JM, Schneider R, Peters AH et al. LSD1 demethylates repressive histone marks to promote androgen-receptor-dependent transcription. Nature 2005; 437: 436–439. 16079795 10.1038/nature04020

[bib31] Adamo A, Sese B, Boue S, Castano J, Paramonov I, Barrero MJ et al. LSD1 regulates the balance between self-renewal and differentiation in human embryonic stem cells. Nat Cell Biol 2011; 13: 652–659. 21602794 10.1038/ncb2246

[bib32] Foster CT, Dovey OM, Lezina L, Luo JL, Gant TW, Barlev N et al. Lysine-specific demethylase 1 regulates the embryonic transcriptome and CoREST stability. Mol Cell Biol 2010; 30: 4851–4863. 20713442 10.1128/MCB.00521-10 PMC2950538

[bib33] Macfarlan TS, Gifford WD, Agarwal S, Driscoll S, Lettieri K, Wang J et al. Endogenous retroviruses and neighboring genes are coordinately repressed by LSD1/KDM1A. Genes Dev 2011; 25: 594–607. 21357675 10.1101/gad.2008511 PMC3059833

[bib34] Wang J, Hevi S, Kurash JK, Lei H, Gay F, Bajko J et al. The lysine demethylase LSD1 (KDM1) is required for maintenance of global DNA methylation. Nat Genet 2009; 41: 125–129. 19098913 10.1038/ng.268

[bib35] Wang J, Scully K, Zhu X, Cai L, Zhang J, Prefontaine GG et al. Opposing LSD1 complexes function in developmental gene activation and repression programmes. Nature 2007; 446: 882–887. 17392792 10.1038/nature05671

[bib36] Zhu D, Hölz S, Metzger E, Pavlovic M, Jandausch A, Jilg C et al. Lysine-specific demethylase 1 regulates differentiation onset and migration of trophoblast stem cells. Nat Commun 2014; 5: 3174. 24448552 10.1038/ncomms4174

[bib37] Whyte WA, Bilodeau S, Orlando DA, Hoke HA, Frampton GM, Foster CT et al. Enhancer decommissioning by LSD1 during embryonic stem cell differentiation. Nature 2012; 482: 221–225. 22297846 10.1038/nature10805 PMC4144424

[bib38] Latos PA, Goncalves A, Oxley D, Mohammed H, Turro E, Hemberger M. Fgf and Esrrb integrate epigenetic and transcriptional networks that regulate self-renewal of trophoblast stem cells. Nat Commun 2015; 6: 7776. 26206133 10.1038/ncomms8776 PMC4525203

[bib39] Luo J, Sladek R, Bader JA, Matthyssen A, Rossant J, Giguere V. Placental abnormalities in mouse embryos lacking the orphan nuclear receptor ERR-beta. Nature 1997; 388: 778–782. 9285590 10.1038/42022

[bib40] Duteil D, Metzger E, Willmann D, Karagianni P, Friedrichs N, Greschik H et al. LSD1 promotes oxidative metabolism of white adipose tissue. Nat Commun 2014; 5: 4093. 24912735 10.1038/ncomms5093 PMC4112219

[bib41] Hino S, Sakamoto A, Nagaoka K, Anan K, Wang Y, Mimasu S et al. FAD-dependent lysine-specific demethylase-1 regulates cellular energy expenditure. Nat Commun 2012; 3: 758. 22453831 10.1038/ncomms1755 PMC3316891

[bib42] Musri MM, Carmona MC, Hanzu FA, Kaliman P, Gomis R, Parrizas M. Histone demethylase LSD1 regulates adipogenesis. J Biol Chem 2010; 285: 30034–30041. 20656681 10.1074/jbc.M110.151209 PMC2943311

[bib43] Maes T, Tirapu I, Mascaro C, Ortega A, Estiarte A, Valls N et al. Preclinical characterization of a potent and selective inhibitor of the histone demethylase KDM1A for MLL leukemia. ASCO Meeting Abstracts 2013; 31: e13543.

[bib44] Huang J, Sengupta R, Espejo AB, Lee MG, Dorsey JA, Richter M et al. p53 is regulated by the lysine demethylase LSD1. Nature 2007; 449: 105–108. 17805299 10.1038/nature06092

[bib45] Mosammaparast N, Kim H, Laurent B, Zhao Y, Lim HJ, Majid MC et al. The histone demethylase LSD1/KDM1A promotes the DNA damage response. J Cell Biol 2013; 203: 457–470. 24217620 10.1083/jcb.201302092 PMC3824007

[bib46] Scoumanne A, Chen X. The lysine-specific demethylase 1 is required for cell proliferation in both p53-dependent and -independent manners. J Biol Chem 2007; 282: 15471–15475. 17409384 10.1074/jbc.M701023200

[bib47] Yang C, Sudderth J, Dang T, Bachoo RM, McDonald JG, DeBerardinis RJ. Glioblastoma cells require glutamate dehydrogenase to survive impairments of glucose metabolism or Akt signaling. Cancer Res 2009; 69: 7986–7993. 19826036 10.1158/0008-5472.CAN-09-2266 PMC2764330

[bib48] Morgan B, Sobotta MC, Dick TP. Measuring E(GSH) and H2O2 with roGFP2-based redox probes. Free Radic Biol Med 2011; 51: 1943–1951. 21964034 10.1016/j.freeradbiomed.2011.08.035

[bib49] Jeong SM, Lee A, Lee J, Haigis MC. SIRT4 protein suppresses tumor formation in genetic models of Myc-induced B cell lymphoma. J Biol Chem 2014; 289: 4135–4144. 24368766 10.1074/jbc.M113.525949 PMC3924279

[bib50] Robinson MM, McBryant SJ, Tsukamoto T, Rojas C, Ferraris DV, Hamilton SK et al. Novel mechanism of inhibition of rat kidney-type glutaminase by bis-2-(5-phenylacetamido-1,2,4-thiadiazol-2-yl)ethyl sulfide (BPTES). Biochem J 2007; 406: 407–414. 17581113 10.1042/BJ20070039 PMC2049044

[bib51] Wang JB, Erickson JW, Fuji R, Ramachandran S, Gao P, Dinavahi R et al. Targeting mitochondrial glutaminase activity inhibits oncogenic transformation. Cancer Cell 2010; 18: 207–219. 20832749 10.1016/j.ccr.2010.08.009 PMC3078749

[bib52] Li C, Allen A, Kwagh J, Doliba NM, Qin W, Najafi H et al. Green tea polyphenols modulate insulin secretion by inhibiting glutamate dehydrogenase. J Biol Chem 2006; 281: 10214–10221. 16476731 10.1074/jbc.M512792200

[bib53] Mathias RA, Greco TM, Oberstein A, Budayeva HG, Chakrabarti R, Rowland EA et al. Sirtuin 4 is a lipoamidase regulating pyruvate dehydrogenase complex activity. Cell 2014; 159: 1615–1625. 25525879 10.1016/j.cell.2014.11.046 PMC4344121

[bib54] Hensley CT, Wasti AT, DeBerardinis RJ. Glutamine and cancer: cell biology, physiology, and clinical opportunities. J Clin Invest 2013; 123: 3678–3684. 23999442 10.1172/JCI69600 PMC3754270

[bib55] Schulze A, Harris AL. How cancer metabolism is tuned for proliferation and vulnerable to disruption. Nature 2012; 491: 364–373. 23151579 10.1038/nature11706

[bib56] Harris WJ, Huang X, Lynch JT, Spencer GJ, Hitchin JR, Li Y et al. The histone demethylase KDM1A sustains the oncogenic potential of MLL-AF9 leukemia stem cells. Cancer Cell 2012; 21: 473–487. 22464800 10.1016/j.ccr.2012.03.014

[bib57] Kahl P, Gullotti L, Heukamp LC, Wolf S, Friedrichs N, Vorreuther R et al. Androgen receptor coactivators lysine-specific histone demethylase 1 and four and a half LIM domain protein 2 predict risk of prostate cancer recurrence. Cancer Res 2006; 66: 11341–11347. 17145880 10.1158/0008-5472.CAN-06-1570

[bib58] Schenk T, Chen WC, Gollner S, Howell L, Jin L, Hebestreit K et al. Inhibition of the LSD1 (KDM1A) demethylase reactivates the all-trans-retinoic acid differentiation pathway in acute myeloid leukemia. Nat Med 2012; 18: 605–611. 22406747 10.1038/nm.2661 PMC3539284

[bib59] Wu J, Hu L, Du Y, Kong F, Pan Y. Prognostic role of LSD1 in various cancers: evidence from a meta-analysis. Onco Targets Ther 2015; 8: 2565–2570. 26451115 10.2147/OTT.S89597 PMC4592051

[bib60] Wada T, Koyama D, Kikuchi J, Honda H, Furukawa Y. Overexpression of the shortest isoform of histone demethylase LSD1 primes hematopoietic stem cells for malignant transformation. Blood 2015; 125: 3731–3746. 25904247 10.1182/blood-2014-11-610907

[bib61] German NJ, Haigis MC. Sirtuins and the metabolic hurdles in cancer. Curr Biol 2015; 25: R569–R583. 26126285 10.1016/j.cub.2015.05.012 PMC5748939

[bib62] Le A, Lane AN, Hamaker M, Bose S, Gouw A, Barbi J et al. Glucose-independent glutamine metabolism via TCA cycling for proliferation and survival in B cells. Cell Metab 2012; 15: 110–121. 22225880 10.1016/j.cmet.2011.12.009 PMC3345194

[bib63] Tanaka S, Kunath T, Hadjantonakis AK, Nagy A, Rossant J. Promotion of trophoblast stem cell proliferation by FGF4. Science 1998; 282: 2072–2075.. 10.1126/science.282.5396.2072 9851926

[bib64] Debacq-Chainiaux F, Erusalimsky JD, Campisi J, Toussaint O. Protocols to detect senescence-associated beta-galactosidase (SA-betagal) activity, a biomarker of senescent cells in culture and *in vivo*. Nat Protoc 2009; 4: 1798–1806. 20010931 10.1038/nprot.2009.191

[bib65] Trapnell C, Roberts A, Goff L, Pertea G, Kim D, Kelley DR et al. Differential gene and transcript expression analysis of RNA-seq experiments with TopHat and Cufflinks. Nat Protoc 2012; 7: 562–578. 22383036 10.1038/nprot.2012.016 PMC3334321

[bib66] Robinson MD, McCarthy DJ, Smyth GK. edgeR: a Bioconductor package for differential expression analysis of digital gene expression data. Bioinformatics 2010; 26: 139–140. 19910308 10.1093/bioinformatics/btp616 PMC2796818

[bib67] Arrigoni L, Richter AS, Betancourt E, Bruder K, Diehl S, Manke T et al. Standardizing chromatin research: a simple and universal method for ChIP-seq. Nucleic Acids Res 2015; 44: e67. 26704968 10.1093/nar/gkv1495 PMC4838356

[bib68] Langmead B, Salzberg SL. Fast gapped-read alignment with Bowtie 2. Nat Methods 2012; 9: 357–359. 22388286 10.1038/nmeth.1923 PMC3322381

[bib69] Zhang Y, Liu T, Meyer CA, Eeckhoute J, Johnson DS, Bernstein BE et al. Model-based analysis of ChIP-Seq (MACS). Genome Biol 2008; 9: R137. 18798982 10.1186/gb-2008-9-9-r137 PMC2592715

[bib70] Robinson JT, Thorvaldsdottir H, Winckler W, Guttman M, Lander ES, Getz G et al. Integrative genomics viewer. Nat Biotechnol 2011; 29: 24–26. 21221095 10.1038/nbt.1754 PMC3346182

[bib71] Heinz S, Benner C, Spann N, Bertolino E, Lin YC, Laslo P et al. Simple combinations of lineage-determining transcription factors prime cis-regulatory elements required for macrophage and B cell identities. Mol Cell 2010; 38: 576–589. 20513432 10.1016/j.molcel.2010.05.004 PMC2898526

[bib72] Giavalisco P, Kohl K, Hummel J, Seiwert B, Willmitzer L. 13C isotope-labeled metabolomes allowing for improved compound annotation and relative quantification in liquid chromatography-mass spectrometry-based metabolomic research. Anal Chem 2009; 81: 6546–6551. 19588932 10.1021/ac900979e

[bib73] Ferrick DA, Neilson A, Beeson C. Advances in measuring cellular bioenergetics using extracellular flux. Drug Discov Today 2008; 13: 268–274. 18342804 10.1016/j.drudis.2007.12.008

