# Supplementary figures and images for: Inactivation of Lsd1 triggers senescence in trophoblast stem cells by induction of Sirt4
Source: Cell Death Dis. 2017 Feb 23;8(2):e2631–. doi: 10.1038/cddis.2017.48 (PMC5386490; doi:10.1038/cddis.2017.48)

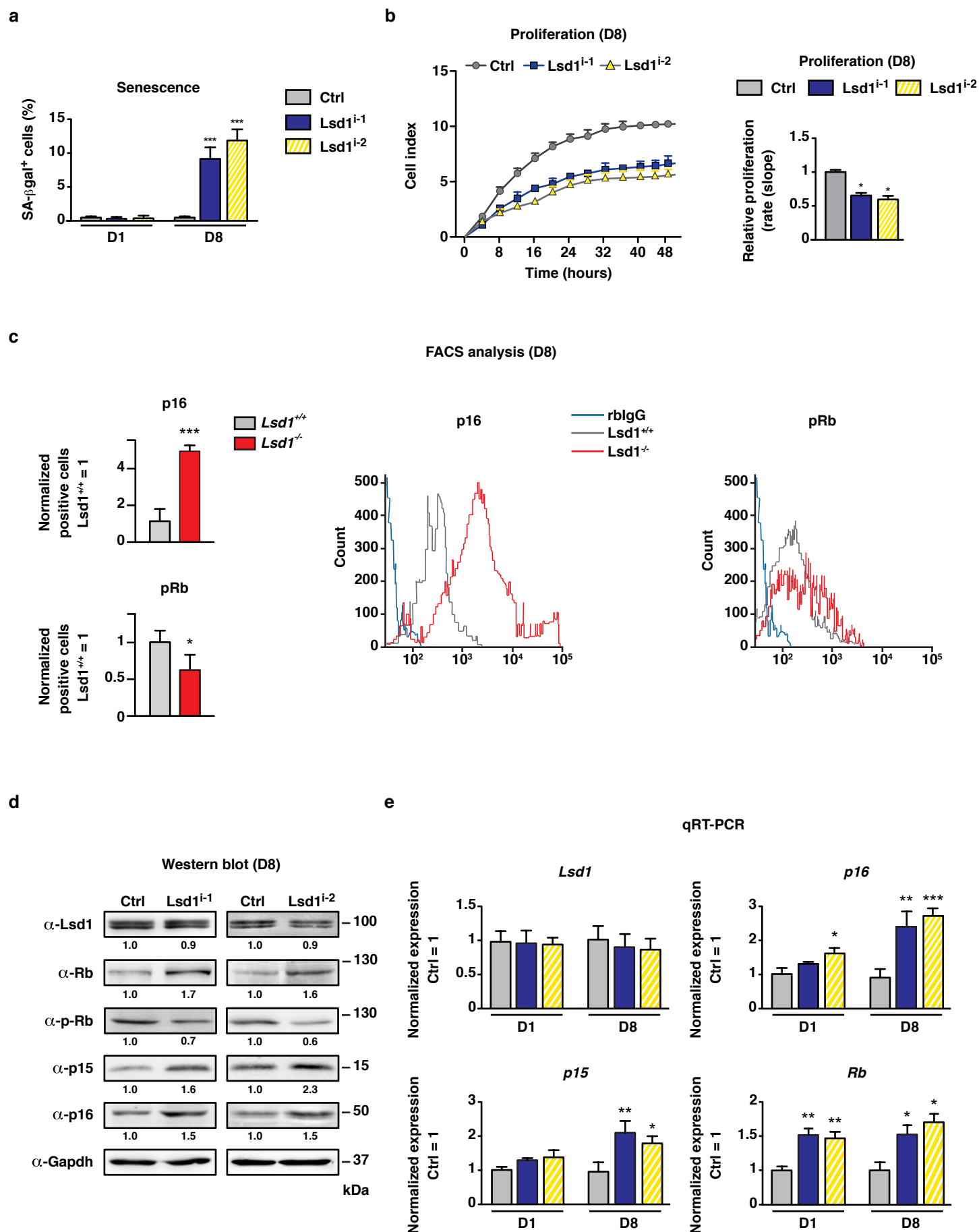

f

## FACS analysis

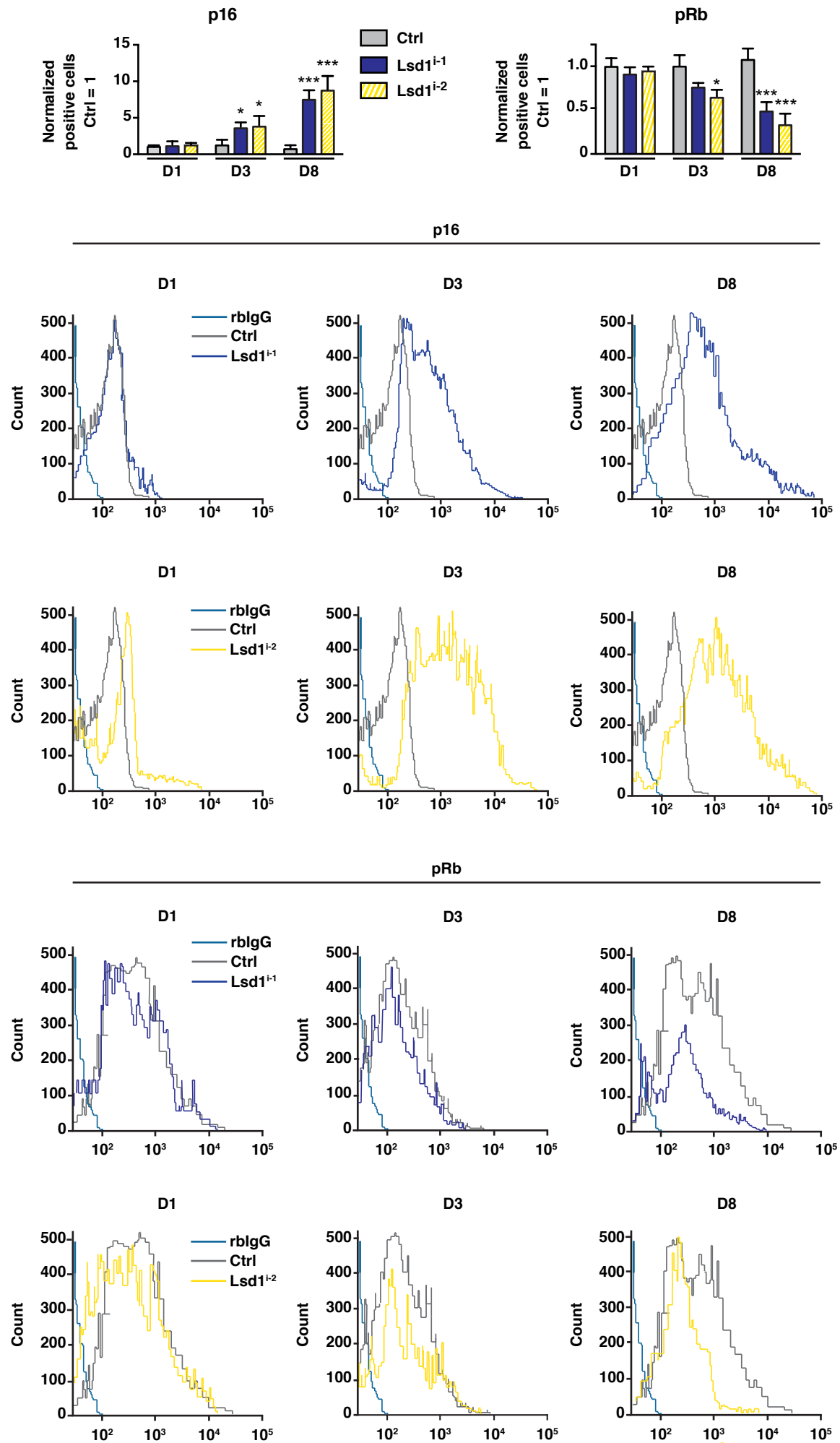

g

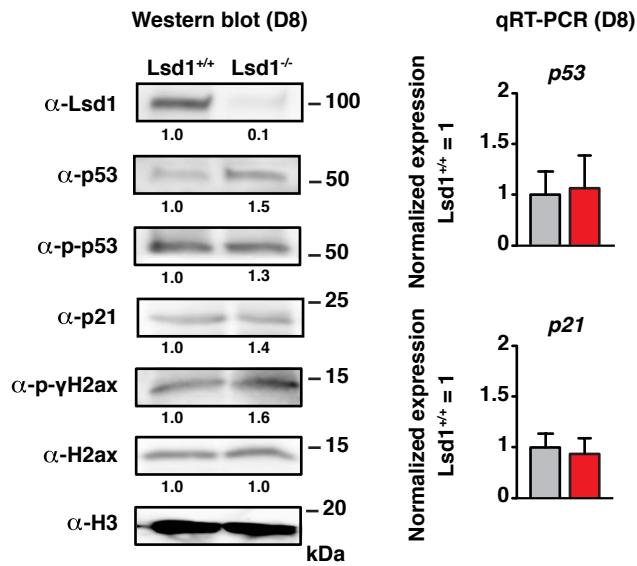

h

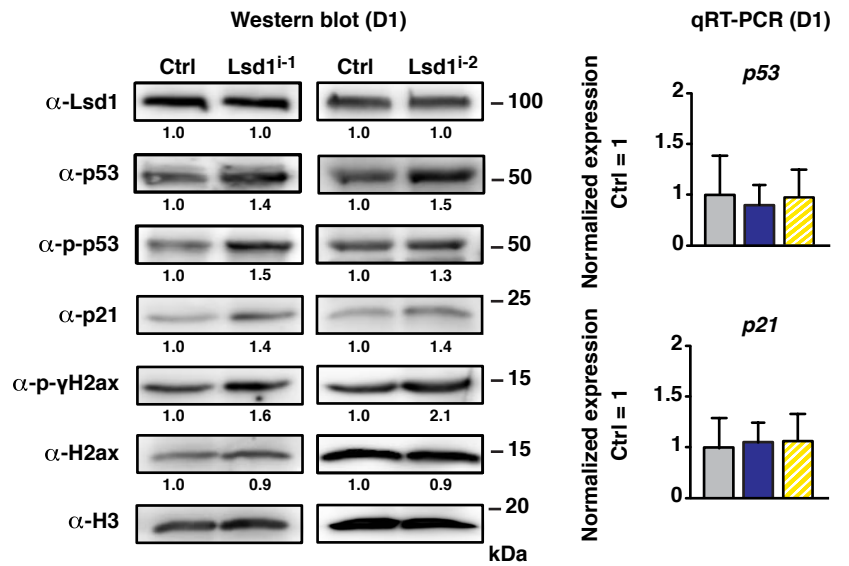

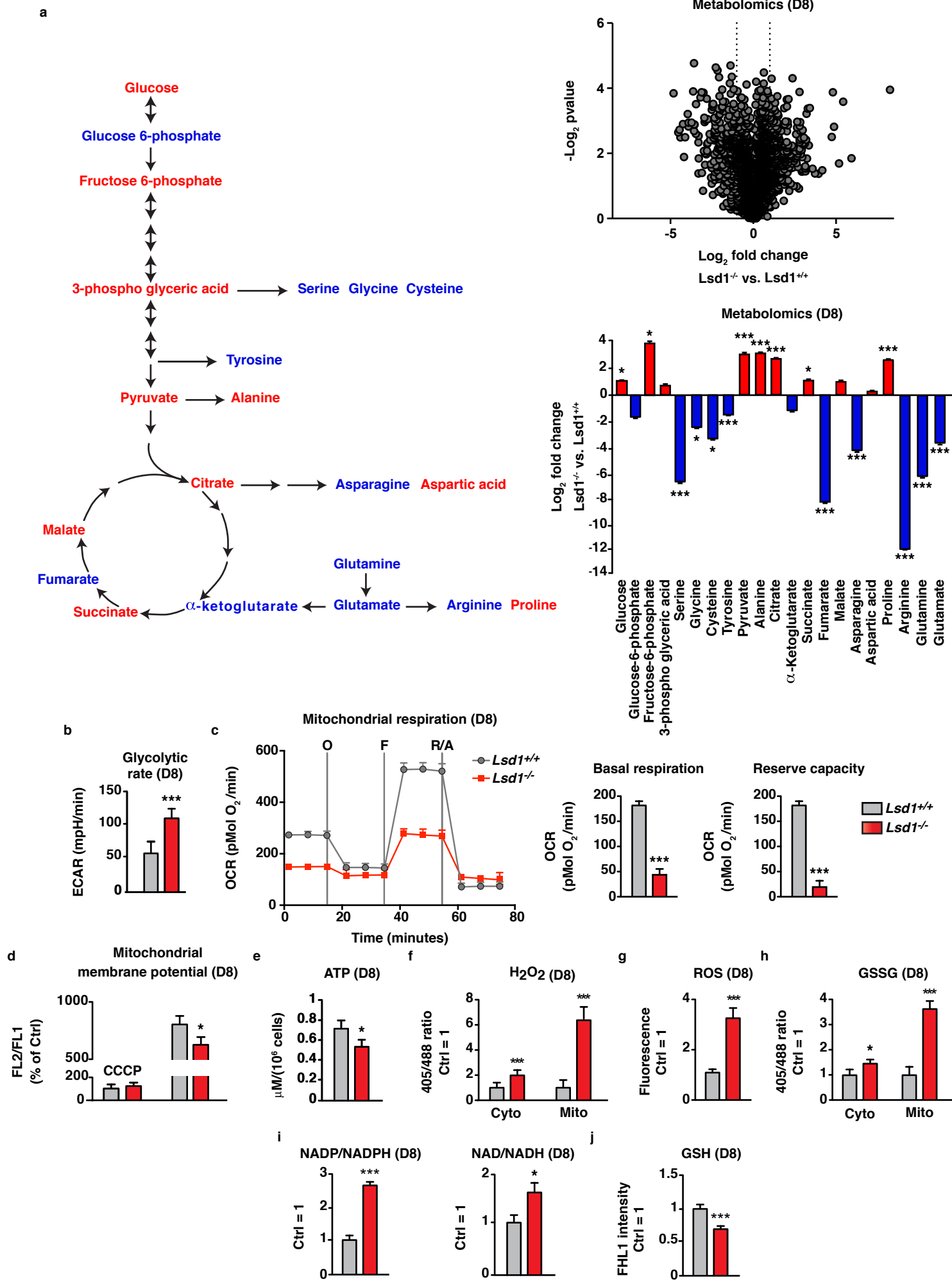

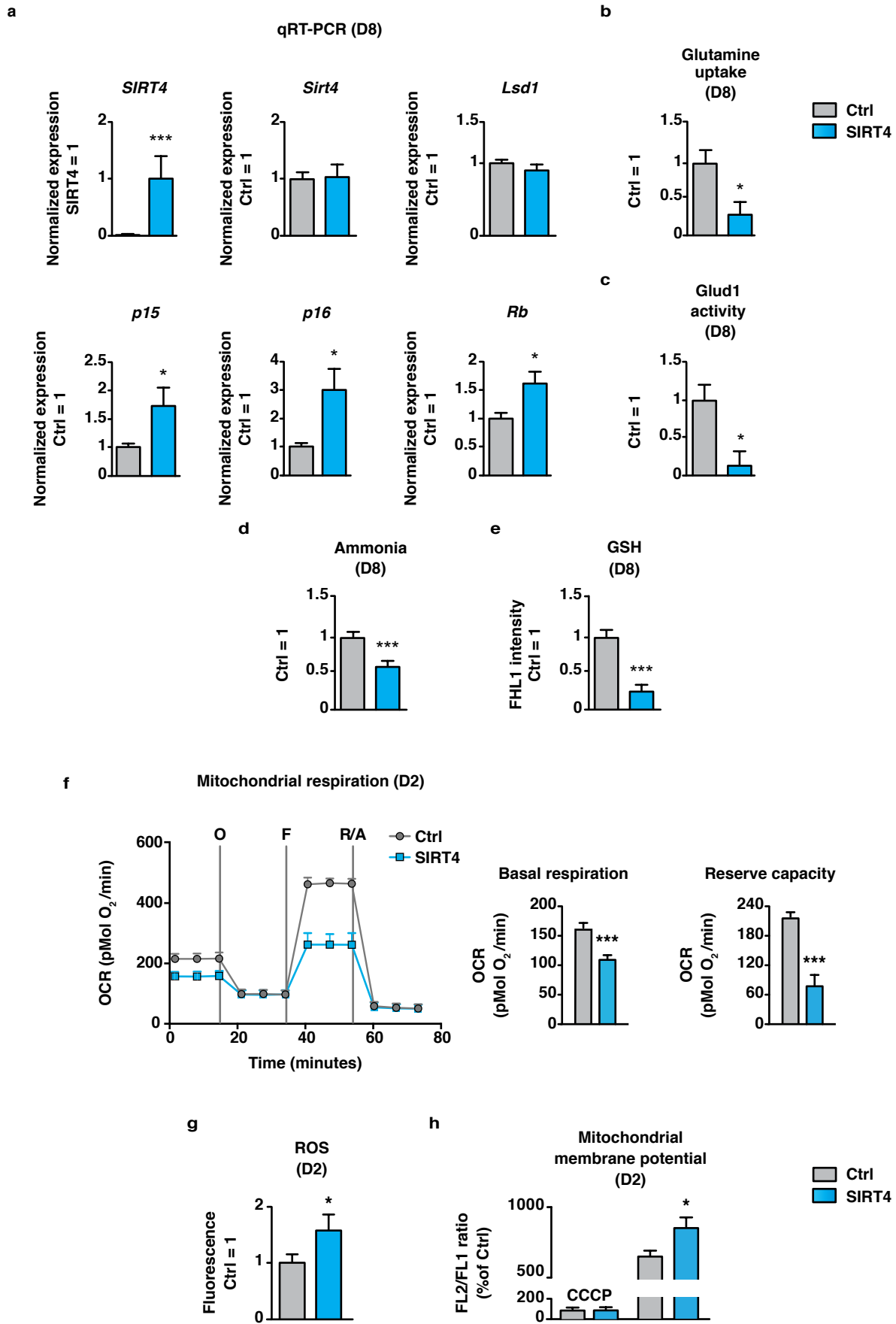

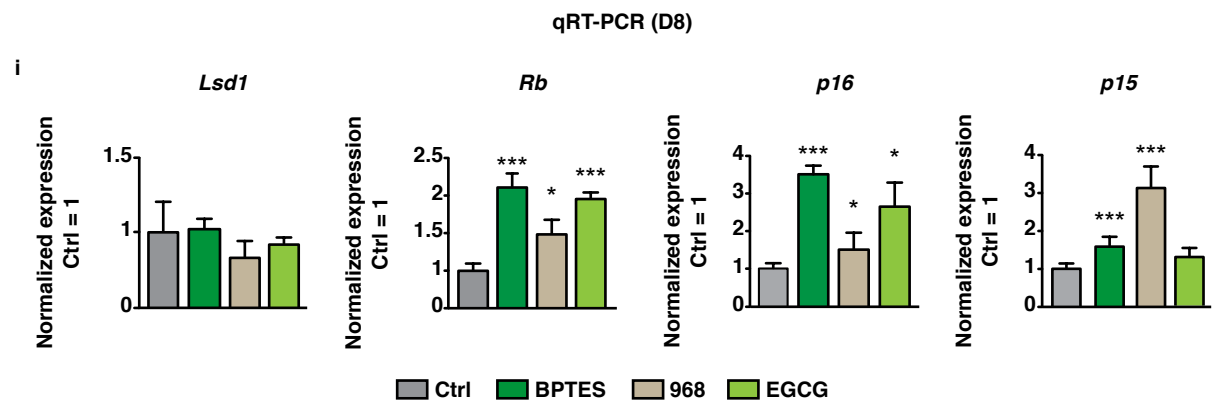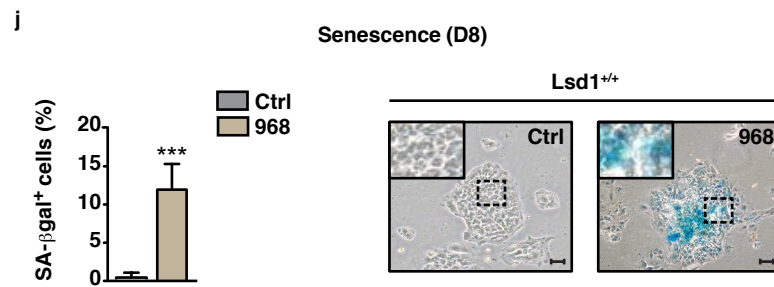

**a**

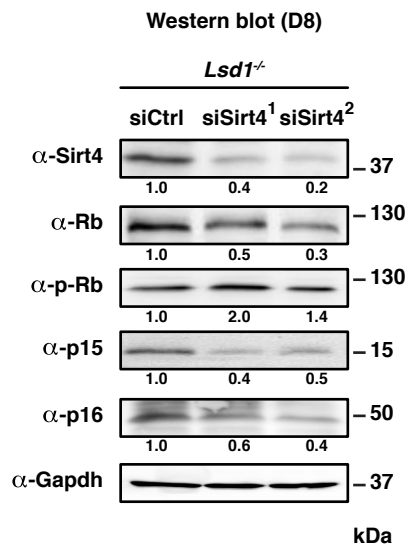

Supplement: Supplementary Figures [file cddis201748x1.pdf]
